# Portable Dynamometer-Based Measurement of Pelvic Floor Muscle Force

**DOI:** 10.1109/JTEHM.2022.3223258

**Published:** 2022-11-18

**Authors:** Batoul El-Sayegh, Chantale Dumoulin, Mohamed Ali, Hussein Assaf, Jacqueline De Jong, Mohamad Sawan, François Leduc-Primeau

**Affiliations:** Department of Electrical EngineeringPolytechnique Montreal 5596 Montreal QC H3T 1J4 Canada; Research CenterInstitut Universtaire de Gériatrie de Montréal Montréal QC H3W 1W4 Canada; School of Rehabilitation, Faculty of MedicineUniversité de Montréal Montréal QC H3N 1X7 Canada; Department of MicroelectronicsElectronics Research Institute 230798 Cairo 12622 Egypt; Physio SPArtos 3800 Interlaken Switzerland; School of EngineeringWestlake University and CenBRAIN Neurotech Center of Excellence, Westlake Institute for Advanced Study Hangzhou 310024 China

**Keywords:** Force measurement, pelvic floor muscle assessment, urinary incontinence, vaginal dynamometer

## Abstract

Objective: In attempts to improve the quality of life of women, continuous projects are sought between rehabilitation intervention and engineering. Using the knowledge of the pelvic floor muscle (PFM) physiology, assessment and training methods are developed to reduce lower urinary tract symptoms such as urinary incontinence. Therefore, this paper covers the design and implementation of a portable vaginal dynamometer. Methods: A PFM probe is designed, 3D printed, assembled, and tested in ten women to assess its acceptability and usability. The feedback from the usability study is used to optimize the PFM probe design. A vaginal dynamometer is developed based on the designed PFM probe, then tested for linearity, repeatability, hysteresis, noise and heat effect, and power consumption. The variability between the different produced PFM probe prototypes is evaluated. Results: Force measurements are made using a load cell. Wireless communication is performed through a Bluetooth low energy transceiver v5.0, with a corresponding interface on both computer and smartphone. The device operates at a 3.3V supply and achieves a power consumption of 49.5 mW in operating mode. Two PFM probe sizes are designed to accommodate different vaginal hiatus sizes, based on usability study feedback. The proposed system allows the physiotherapist to wirelessly monitor variation in pelvic floor muscle force during assessment and/or training. Discussion/Conclusion: The testing results showed that the newly designed system has the potential to measure the PFM function in functional conditions such as the standing position.

## I. Introduction

Involuntary loss of urine (urinary incontinence) is a highly prevalent condition, especially among women. Approximately, one in three women suffers from this condition [1]. In 2012, the Canadian Urological Association estimated that 3.3 million Canadians suffer from urinary incontinence (UI). In addition to stress and social isolation, UI affects a patient’s self-esteem and quality of life [2]. Furthermore, a financial burden of high significance is imposed on patients and healthcare organizations. The expenses are estimated to be 16.4 billion dollars annually according to a study made on a US population [3]. UI in women is linked to defects and/or dysfunctions in the pelvic floor muscle (PFM), which is responsible for supporting the bladder neck and closing the urethra. The assessment of PFM forces evaluates the capacity of PFM to contract and relax actively. The first line of treatment for UI is PFM rehabilitation [4]. This treatment has been shown to improve pelvic organ support, and urethral closure, thus preventing urinary leakage. It has been recommended to assess the PFM function prior to and during PFM training to evaluate dysfunctions, give the best training program for specific PFM dysfunction, and measure improvements [4].

Proper assessment of PFM is considered a key element in the management of UI in women. Several measurement methods have been developed to assess and train the PFM [5], [6], [7], [8]. The most common assessment method is digital palpation, where the physiotherapist inserts his/her fingers in the vagina of the patient to palpate the PFM in order to feel passive forces at rest and active forces during maximal PFM contraction. However, repeatability is limited as it is a subjective measurement of PFM function [5] and is dependent on the evaluator’s competency. Perineometry, electromyography (EMG), and imaging techniques (ultrasound and magnetic resonance imaging (MRI)) are indirect force measurement methods [6]. The major drawback of EMG is the possible bias of the PFM measurements due to cross-talk from the surrounding muscles. As for the perineometer (pressure measures), the validity of the measurements can be affected by artifacts such as intra-abdominal pressure.

Over the last 15 years, dynamometry has been proposed to achieve reliable and direct measurements [4], [9]. This device is inserted into the vagina, to assess the PFM function, through measuring the PFM resting and contractile forces using strain gauges (SG) mounted on a speculum. Then, a processing unit is used to process the force data. Finally, the data is displayed on a PC monitor. Several dynamometer prototypes, for research purposes, have been presented in the literature [6], [9]. However, in these approaches, the force signal is transferred to a user interface, for processing and display, through a wired connection between the user interface and the processing unit. The presence of a wire between the processing unit and the computer is problematic for both the patient and therapist in the evaluation and treatment environment. Moreover, and based on the system design, the wire could limit the measurement position to a supine position rather than a standing position in which UI is much more prevalent.

**FIGURE 1.**
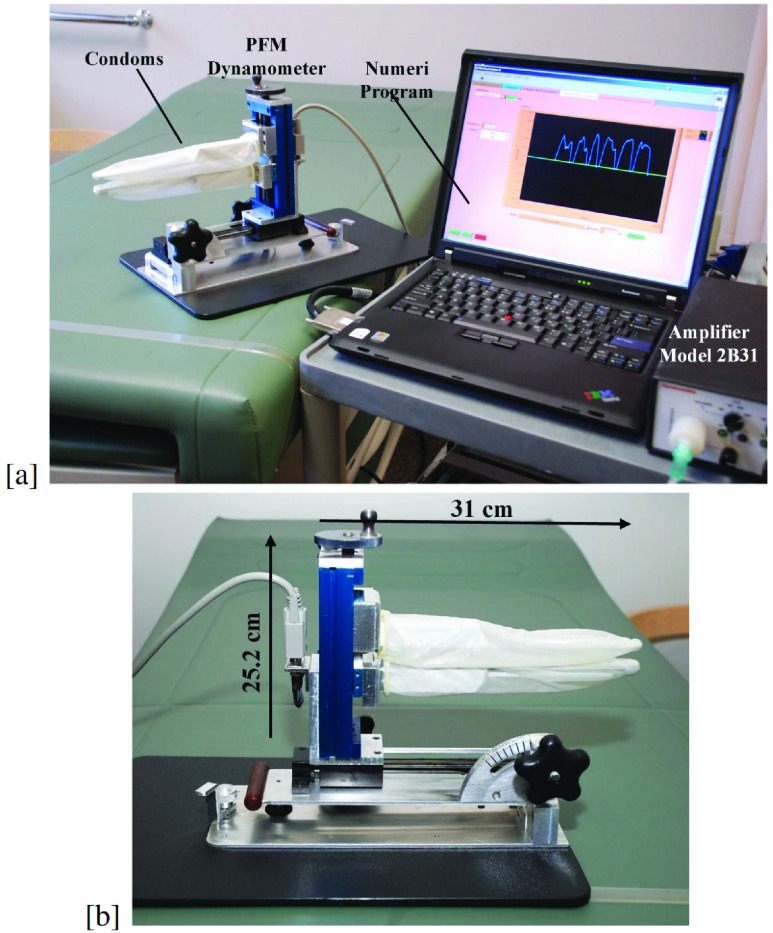
Example of a wired dynamometer-based measurement system: (a) Complete research system used at Centre de recherche de l’Institut universitaire de gé riatrie de Montré al (CRIUGM), and (b) Experimental prototype.

An example of a wired PFM real-time measurement system used in the Incontinence and Aging Laboratory at Centre de recherche de l’Institut universitaire de geriatrie de Montré al (CRIUGM) is depicted in Fig. 1(a), where a dynamometer is used to measure the PFM force and transfer it to the amplifier model (Analog device model 2B31). Then, the amplified signal is delivered to a laptop computer for further processing and display. In addition to system complexity and requiring special training to be used, the employed dynamometer (developed by Dumoulin et al. [10] and improved by Morin et al. [11]) is bulky, and heavy, as shown in Fig. 1(b). Although it is adequate for research, it is not optimal for clinical applications.

Recognizing the importance of a portable direct PFM force measurement tool in clinical research and rehabilitation intervention, our long-term objective is to build an electronic device that is wearable, and wirelessly operable which includes a biofeedback real-time measurement interface. In this system, the processing and the wireless transmission will be designed with a small, printed circuit board so that it can be included in the Dynamometer. Therefore, no wires will be coming out from the vagina.

**FIGURE 2.**
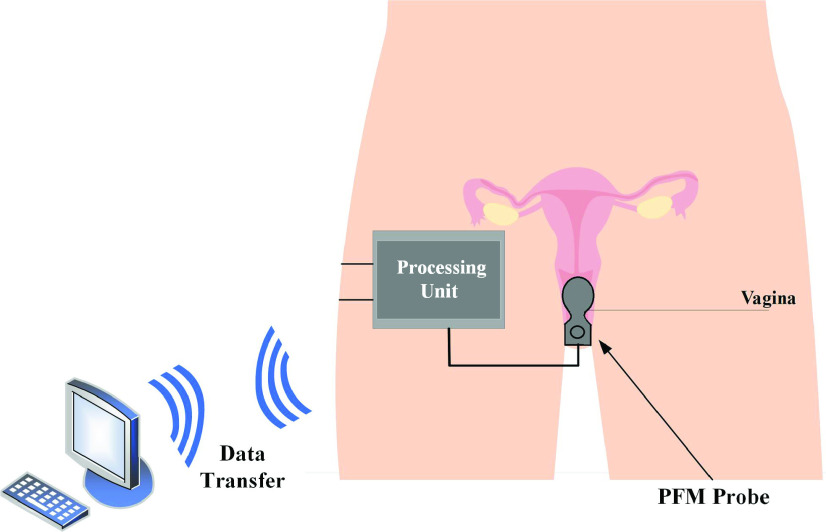
Overview of the proposed portable vaginal dynamometer.

A proof-of-concept prototype, for a system measuring pelvic floor muscle forces, based on a previous dynamometer, has been developed and tested in an earlier study [12]. Further, and as a first step towards achieving the projected system, a complete original portable vaginal dynamometer prototype is given in this paper. The vaginal dynamometer consists of a newly designed PFM probe, used to acquire the force signal, connected to a processing unit that has been constructed from discrete components. An overview of the proposed system is illustrated in Fig. 2.

The proposed system is designed to be used in research and in rehabilitation settings. In research settings, the system is planned to be used in clinical research aiming to better understand the pelvic floor muscle function and dysfunction and to evaluate the effect of UI treatments on the pelvic floor muscles in functional positions such as standing position. In rehabilitation settings, the system will provide biological feedback during PFM training, while providing flexibility and portability for both the participant and the physiotherapist.

The presented system in this paper is a part of a developed geriatric rehabilitation exergame that uses wearable sensors (vaginal dynamometer and shoes step detection) and a web-based interface for the treatment of UI (AAL VITAAL project (https://www.aal-europe.eu/projects/vitaal/)). With a portable vaginal dynamometer, which allows women to move freely while exercising, older adults with mobility limitations, cognitive impairment, and urinary incontinence can train their strength, balance, and cognitive skills at once with such exergames.

The paper is organized as follows: the new PFM probe design is presented in Section II. Section III presents the architecture of the proposed system, where the function of the various building blocks is described. Then, the experimental results of the implemented system are presented in Section IV and discussed in Section V. Conclusion and future work are given in Section VI.

## II. Pelvic Floor Muscle Probe Design

Presently, there is no gold standard or universal reference for PFM measurement. Therefore, designing a reliable and valid assessment tool to both measure and train PFM is of high importance. We describe in this section, the literature review supporting the proposed PFM probe design in terms of the type of sensor used and the sensor’s location, the direction of measurement, and finally the dimensions and shape.

### A. PFM Probe Sensor Type

As the aim of the newly developed system is to directly measure the PFM resting and contractile forces, direct force sensors have been considered such as strain gauges (SG), load cells, and force-sensitive resistors (FSR). Among the force sensors, a load cell is implemented due to its flexibility in the design integration, and easy installation, allowing for rapid prototyping.

### B. PFM Probe Sensor Location

Several works have investigated the force distribution in the pelvic floor under different pelvic floor muscle activities [13], [14], [15]. Such studies aimed at identifying the critical dimensional parameters for sensor measurements. For this reason, different studies have used different approaches to present the different dimensional parameters such as the location of force inside the vagina, vaginal length, vaginal width, and direction of measurement. These studies aimed at identifying the optimal strain gauge sensor’s location on the PFM probe.

In 1992, a study described measuring vaginal pressure with a 1.5 cm balloon device. The women performed three PFM contractions with a vaginal balloon placed in four different positions: 1) against the vaginal vault and in the posterior fornix, 2) in the proximal upper third of the vagina, 3) with the middle of the balloon 3.5 cm from the introitus vagina and 4) with half of the balloon outside the introitus vagina. The study found the highest pressures were recorded when the middle of the balloon was placed 3.5 cm from the vaginal introitus [13].

In 2005, a rapid pull-through technique utilizing a four-channel water-perfused catheter on a motor-driven puller was used in a study to create a pressure profile in the vaginal cavity for each woman. The profiles were measured with the women at rest and during a sustained contraction of the levator ani muscle. The individual subject’s pressure profiles were averaged to create a composite profile at rest and during squeeze. The study made sure to cover the mid-portion of the vaginal canal for all participants (around 3.5 cm from the vaginal introitus). The study concluded that around 3.5 cm from the vaginal introitus is the most relevant region for pelvic floor muscle contraction, confirming previous results [14].

In 2017, a study used a novel instrumented probe for measuring 3D pressure distribution along the vaginal canal. The probe consisting of a hard plastic cylinder was covered by capacitive transducers placed in a 10 by 10 matrix configuration. The pressure distribution was constructed during two activities (maximum contraction and Valsalva maneuver). Through examining the 3D pressure with 100 sensors, the study showed that the maximal pressure values were located between 3 and 4 cm from the vaginal opening [15].

Therefore, since the pelvic floor muscular mass is located around 3.5 cm from the vaginal cavity opening, the SG sensors are arranged such that 3.5 cm from the vaginal introits is at the midpoint. This arrangement allows the sensors to record the optimal PFM measurements.

### C. Direction of Measurement

The most challenging part of the design process is the high-performance requirements expected from the system in terms of repeatability, validity, and sensitivity of measurements. As a part of the validity of the system, there is a critical need to differentiate what is PFM contraction and what is not. This is because, when women are verbally taught how to contract their PFM, around 40% of women do an incorrect or a reverse contraction or a push which weakens their PFM rather than strengthens it [16]. Thus, it is very important to understand how to differentiate between correct and incorrect PFM.

Considering the functional anatomy of the PFM, a ventral-cephalic contraction pattern is observed (compressing the vagina, urethra, and rectum against the pubic bone) during a correct PFM contraction. Whereas upon an incorrect or reverse contraction of the PFM also called the Valsalva maneuver, a dorsal-caudal pattern is observed.

Clinically, and in research measurements of the active/ maximal PFM forces are obtained by a digital or by an instrument measuring force exerted by the PFM in the anteroposterior direction in the inclined vagina (ventral-cephalic contraction pattern). These measurements are conducted with women with UI, and sexual dysfunctions and have been shown to produce larger pressures and force changes in anteroposterior than in latero-lateral [4], [17], [18], [19].

It is important to understand the movements of the PFM muscles to design the PFM probe. For example, the importance of identifying the direction of measurement of the PFM probe is that the configuration of the sensors is affected by the axis of measurement.

Based on the presented information, it could be expected that our proposed vaginal dynamometer is focused on the anteroposterior assessment of the forces inside the vaginal cavity.

### D. PFM Probe Dimensions and Shape

The dimensions and shape of the PFM probe are two critical parameters in the design process. The challenge in these two parameters arises from the quantitative interindividual differences in vaginal morphology which can be related to the presence or not of vulvar pain, sexual activity, vaginal delivery, aging, and menopause. Further, limitations in terms of the area are imposed from the PFM probe possible dimensions. Clinically, the major concerns when designing the dimensions and shape of the PDM probe are as follows:

1) What are the optimal probe dimensions that can fit different vaginal sizes and morphologies for different women’s age groups? Considering that the vaginal length reduces with age [20].
2) How can the probe stay stable inside the vagina while exercising in both standing and supine positions?

Several limitations and suggested dimensions have been reported in the literature. Firstly, the muscular mass of the pelvic floor is located some 3.5 cm from the opening of the vaginal cavity [13]. Thus, the depth of the PFM probe, that is the portion entered into the vaginal cavity, is suggested to be more than 3.5 cm so that the force of the PFM is well captured [10], [13], [15]. Further, there should be some extra length to make sure the PFM probe stays stable inside the vagina. However, as a limitation and to consider young and elderly women the maximum depth is about 7 cm [20]. Therefore, a 6- cm depth allows the peri-vaginal portion of the pelvic floor to squeeze on the PFM probe while the PFM probe presses underneath the pubic bone to provide stability. Secondly, an aperture range (opening of the dynamometer) between 3 to 4 cm has been proven to have good reliability for PFM assessment measurement and gave better results and more trustworthy measurements of PFM force [15], [21].

### E. Proposed PFM Probe Design

**FIGURE 3.**
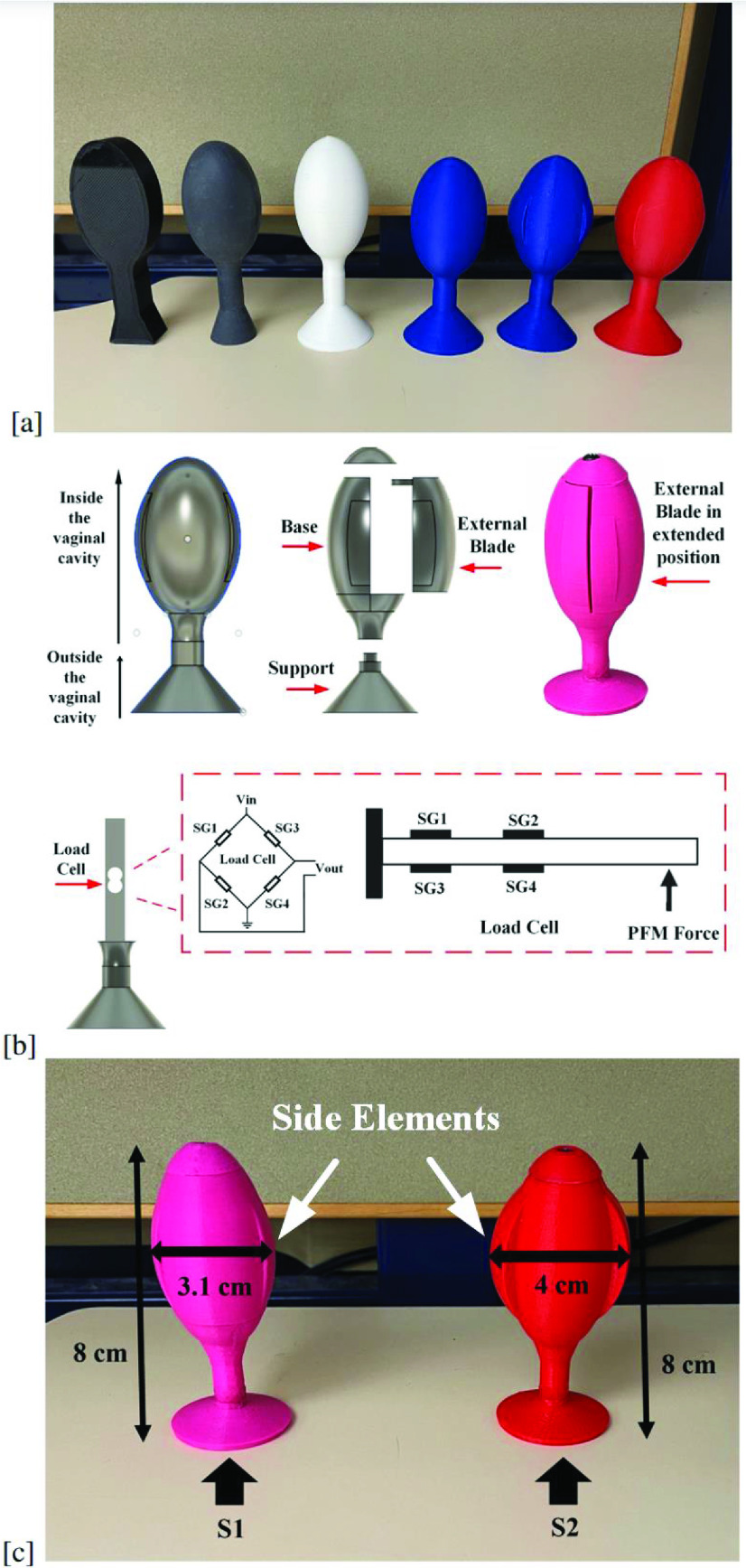
Proposed PFM probe design*: (a) Different PFM probe 3D printed prototypes, (b) PFM probe CAD design, (c) PFM probe final design with the two sizes available (S1 S2). * The PFM probe is connected to the processing unit.

Taking into account all the previously mentioned criteria, a complete PFM probe design is proposed. Three-dimensional (3D) models of the PFM probe were designed according to the detailed requirements and limitations presented in terms of length and width (L = 6.5cm inside the vaginal cavity; D = 3.1 cm). Then the models were 3D printed using Polylactic acid and sanded to assure the maximum smoothness required for the mold painting step. Each time a model was printed, it was tested for usability by 2-3 women. After six enhancements the optimal prototype was reached. The design enhancements targeted enhancing the proportionality between the different probe parts and adding side elements for more stability inside the vaginal cavity. The different 3D-printed prototypes are shown in Fig. 3(a).

Figure 3(b) shows the final PFM probe design. The proposed PFM probe is composed of three major parts: support, base, and external blade. The support can be analogized to the tail of the PFM probe. The support remains at the entrance of the vagina. The external blade is movable, fixed from one side to the PFM probe, and free from the other end. A load cell, utilizing the principle of a cantilever beam, is used as the base.

The SG mounted on the load cell utilizes a full Wheatstone bridge configuration using differential arrangement, as can be seen in Figure 3(b). This arrangement ensures that the PFM force is constant wherever the force is applied on the length of the dynamometer. The differential arrangement has been used previously by our team and in similar systems (Dynamometer for upper limb [22] / Dynamometer for hip and knee [23]).

The PFM forces exerted on the PFM probe induce a strain on the external movable blade, causing the blade to move inwards. The strain is then transmitted to the load cell. The load cell measures the strain through a change in the load cells’ electrical resistance. The resistance change, in turn, is measured as a voltage variation. Through the calibration process, a calibration factor is obtained. The output voltage values of the PFM probe are then converted into units of force using the calibration factor.

The PFM probe prototype shown in Fig 3 (b) promotes stability inside the vaginal cavity through its unique design. The side elements situated on the left and right surfaces of the PFM probe are designed to prevent the PFM probe from turning. The PFM probe has been primarily tested for comfortability by four of our expert physiotherapists.

### F. PFM Probe Biocompatible Prototype

To ensure the biocompatibility of the PFM probe, medical-grade silicone is used as an outer protective cover. The medical grade silicone grade is made up of a specific thickness of silicone mixture (Factor II A-103 Medical grade elastomer 1 lb kit). The silicone mixture consists of a base and a curing agent. The mix ratio is 10:1; every 10 grams base with a 1-gram curing agent. The thickness of the cover layer used depends on the stiffness needed for the specified application. Increasing the thickness of the cover layer increases the stiffness, thus making it less compliant. In our application, the thickness is chosen to be 1 mm, as a compromise between the stiffness and compliance needs of the system. After making the silicone mixture, the mixture is placed for around 15 minutes in a vacuum pump to remove air bubbles created upon mixing. Then using a paintbrush, the silicone mixture is applied and pressed on the PFM probe. Then the PFM probe is placed in a rotating oven to dry at a speed of 66 revolutions per minute.

### G. Vaginal Dynamometer Usability Study

As pelvic floor muscle training (PFMT) is considered the first-line treatment of UI [1], a geriatric rehabilitation exergame that uses wearable sensors (vaginal dynamometer and shoes step detection) and a web-based interface is developed by an international research collaboration, from Belgium, Canada, Portugal, and Switzerland, for the treatment of UI [24]. Exergames are video game interactions that request the player to move to play the game and to be physically active [25].

A usability study including ten women (five from Switzerland and five from Canada) aged 65 and over with urinary incontinence was conducted to assess the acceptability, game experience, and usability of the developed exergame. During the study, the participants tried the vaginal dynamometer as a part of the exergame in a 30-minute session, in a standing position. More specifically, the vaginal dynamometer was considered as a PFM sensor and was used to advance the game. One of the targets of the exergame was pelvic floor muscle training. The usability of the vaginal dynamometer was assessed during the game using the think-aloud method [26] and after the exercise through a post-interview. The results of this usability study were used to identify problematic issues and make recommendations to optimize the vaginal dynamometer design.

During the think-aloud assessment and post-interview participants experienced mixed positive and negative impressions about the vaginal dynamometer. Although all participants liked the exergame and experienced ‘joy’, ‘fun’, or ‘happiness’ while playing the game (n = 10; 100%), three major issues were identified based on the participants’ feedback. First, the PFM probe body and support were disassembled easily upon pulling out the PFM probe from the vagina. Secondly, some participants tended to pull the PFM probe from the connecting wire joining the PFM probe to the processing unit. This occurred at the end of the session when the participants were not sure how to remove the PFM probe. The pulling forces damaged the adhesion of the wire to the PFM probe. Lastly, some women experienced discomfort (40%; n = 4) or were not able to use it due to the dynamometer displacement (20%; n = 2) during the game as the PFM probe was too small for their vaginal cavity.

To overcome the problems, the PFM probe design was altered. The lock mechanism of the PFM probe was changed from push-fit to welding the two parts together after assembly. The welding of the support to the body increased the probe’s stiffness and ensured the resistance of the support to different women pulling forces. Further, and as some women tended to pull the PFM probe out from the connecting wire, part of the connecting wire was partially embedded inside the PFM probe body and adhered with a strong glue that blocks any possible wire separation. Finally, two PFM probe sizes (S1 S2) were developed to overcome the discomfort caused by the dynamometer displacement. Both sized probes have the same length ( $\text{L}=8$ cm), however, the diameter is different (Size1 (S1): $\text{D}=3.1$ cm and Size2 (S2): $\text{D}=4$ cm respectively). The two sizes allow to accommodate different vaginal hiatus sizes.

Figure 3(c) shows the final PFM probe design, with the two sizes available, used in the proposed vaginal dynamometer.

## III. Portable Vaginal Dynamometer Implementation

**FIGURE 4.**
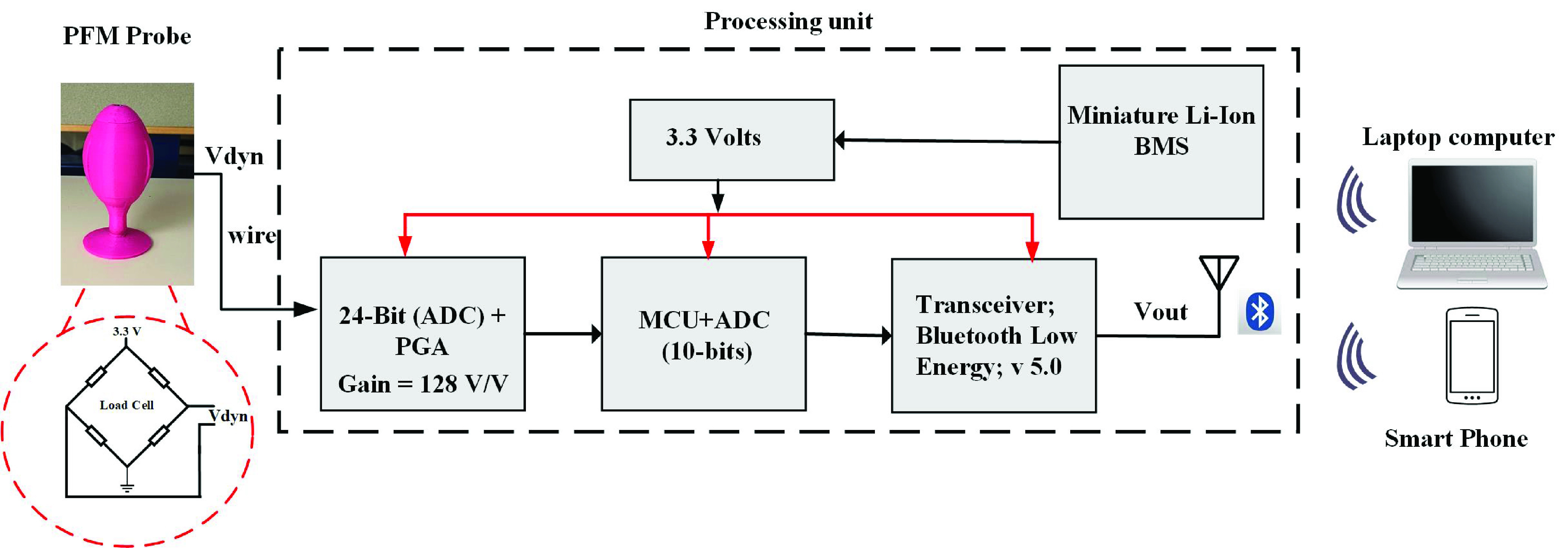
Block diagram of the proposed portable vaginal dynamometer intended to collect force variation and send it to a local base station for clinical application (Laptop computer including RF receiver/ Smartphone).

Figure 4 presents the block diagram of the proposed portable vaginal dynamometer intended to collect force variation and send it to a local base station (Laptop computer including RF receiver/ smartphone). The portable vaginal dynamometer is composed of three major building blocks: the PFM probe, the processing unit, and the graphical user interface (GUI). The PFM probe is connected through a wire to the processing unit. The processing unit, on the other hand, sends the data wirelessly to the GUI. The PFM probe voltage variations are inputted to the processing unit, where the programmable gain amplifier (PGA) amplifies the signal so that the signal amplitudes are adequate for digitization by the 24-Bit analog-to-digital converter (ADC). The Micro-Controller (MCU) then reads the digitized signal, calibrates it, and sends the calibrated signal to the Bluetooth module. Finally, the Bluetooth module transmits the processed data to the chosen GUI.

In the following subsections, the processing unit components along with their specifications are presented in detail.

### A. MCU

The Arduino Pro Mini 328- 3.3V/8 MHz board, which comprising of both MCU and ADC, has been considered in our design due to its compact size (18 mm x 33 mm), lightweight (≤2 g), thinness (0.8 mm thickness), low-power consumption, and low cost (8-9 USD dollars) compared with other off-the-shelf MCU boards. The board uses the ATmega328 microcontroller and provides fourteen digital inputs/outputs, eight analog inputs, and an 8-MHz crystal oscillator Arduino Pro Mini has 6 internal ADCs of 10 bits each. The ADCs are connected internally to the analog input. The MCU supply voltage can be provided using an external power source, such as a battery. A 3.3 V voltage regulator is available on board, allowing a wide range of input voltage (3.3-12 V DC). An Atmega16U2-based USB-to-Serial converter of regulated 3.3 V is required to connect the Arduino pro mini to the computer for further programming. The Arduino has a corresponding software Arduino IDE which includes different programming languages. Arduino IDE is also compatible with MATLAB and LabVIEW, and the data can be imported to other real-time curve plotting applications such as Meguno LINK.

### B. 24 Bit Precision Analogue to Digital Converter

The employed ADC (HX711) is a precision 24-Bit ADC module with two selectable differential input channels. It operates in a voltage range between 2.6 and 5.5 V and consumes a current of less than (1.5 $\mu \text{A}$) in operating mode, and less than (1 $\mu \text{A}$) in power down. An on-chip active low-noise PGA with selectable gain of 32, 64, and 128 is also utilized.

### C. Bluetooth Module

CC2640R2F Bluetooth low energy (BLE) wireless MCU chip from Texas instrument implemented in DSD TECH HM-19 module has been used as a transceiver to send the digitized force signal to the Arduino interface (operated on a laptop computer) or/and Phone app for both Android and IOS (DSDTECHBluetooth). The wireless communication protocol used is Bluetooth low energy version 5.0. HM-19 can be used as both master or slave and operates within a supply range of 1.9 V to 3.7 V. Also, it features an open communication range of up to 100 m, which is sufficient for our application. To minimize the power consumption, the HM-19 module operates in two power modes; active and sleep, which helps in decreasing the power consumption of the complete system.

### D. Power Supply

Taking into account the power supply operation range of the MCU (3.3V- 12V), ADC (2.6V-5.5V), and BLE module (1.9V-3.7V), the need for a rechargeable battery to overcome the complexity of changing the battery in an international research project, a 3.7V lithium-ion battery has been chosen. The battery height was the most critical constraint in our design, which controlled the size of the battery chosen. The length of the battery is 5.1 cm, the width is 3.4 cm, and the height is 0.6 cm. The flat design of the lithium-ion battery allows for overcoming any possible size limitations.

### E. Power Management Design

As lithium-ion battery fluctuates between 4.2V (when the battery is fully charged), and 3.3V (when the battery is drained off), battery management is essential to monitor the voltage fluctuations of the battery, and to allow for recharging the battery. MCP73831/2 linear charge management controller from Microchip implemented in Adafruit micro-lipo battery charger has been used, which features a 100 mA or 500 mA charge current.

Due to the lack of the specific size required, a box has been designed and 3D printed specifically for our application. The box takes into consideration the stability of the PCB and includes a case for the lithium-ion battery.

## IV. Experimental Results

The main interest of this portable vaginal dynamometer is its capability to assess and train the PFM in women with lower urinary tract dysfunction, in this case, UI, in the standing position, which is the naturally occurring position of UI.

The vaginal dynamometer is supplied with a 3.3 V supply. The gain and sampling frequency are chosen to be 128 V/V, and 20 Hz respectively. Further, the ability to modify the sampling rate allows for fast and easy integration of the system data to other possible devices such as an exergame or mobile application. In addition, the system can be easily adjusted to accept other different force/pressure sensors. The properties of the system are detailed in Table 1.

**TABLE 1:** Measurement Features for the Vaginal Dynamometer

Parameter	Measured Value
Power Supply	3.3 V
Power consumption	< 49.5 mW
Device weight	44.5g
PCB Dimensions	$5.46 \times 4.57 \text{ cm}$
Data Transmission	Bluetooth v5.0 BLE
Sampling (per second)	20 samples

**FIGURE 5.**
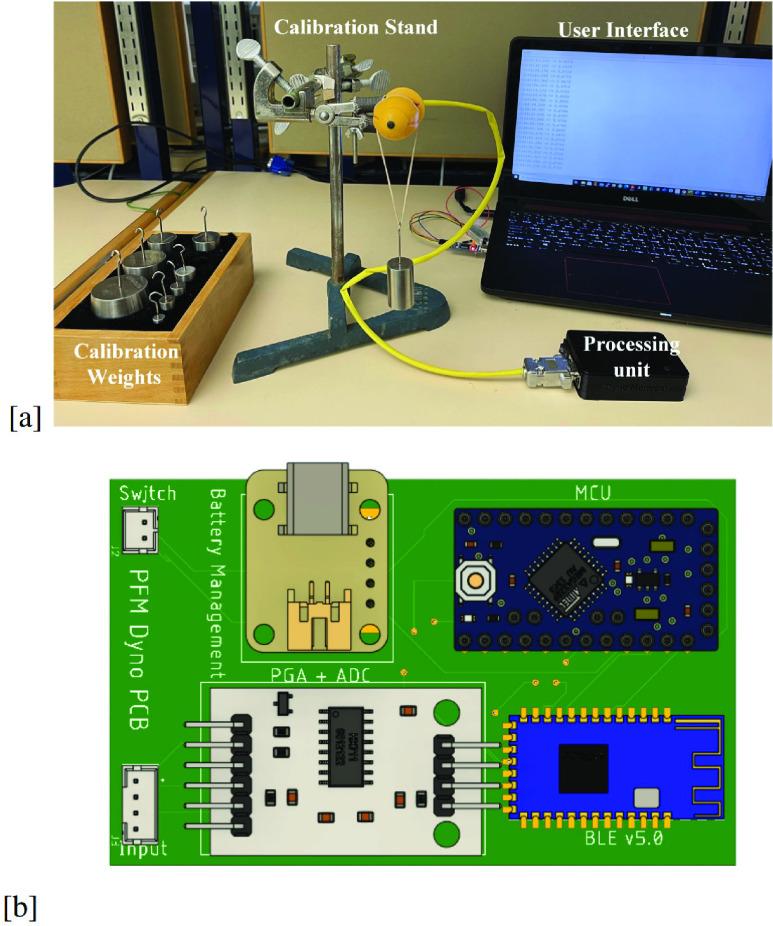
(a) Vaginal dynamometer test bench photograph, and (b) PCB design of the processing unit.

The proposed vaginal dynamometer hardware has been realized and tested using the test bench shown in Fig 5(a). Firstly, each building block of the system is tested separately for proper functioning and characteristics fulfillment. Then, the complete system is validated with the Arduino IDE interface developed for results visualization.

The vaginal dynamometer was first calibrated to obtain the calibration factor that converts to force (N). Also, the system has been assessed for linearity, repeatability, hysteresis, accuracy, noise and heat effect, and power consumption.

### A. Calibration

The vaginal dynamometer is calibrated before proceeding with plotting real-time force curves. Calibration is needed to obtain the conversion factor to force (N).

**FIGURE 6.**
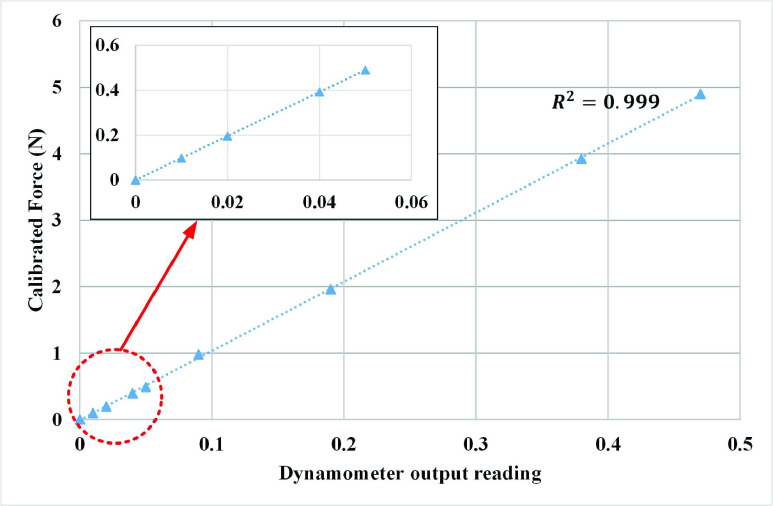
Vaginal dynamometer calibration graph.

Previously known 8 calibrated weights (10g, 20g, 40g, 50g, 100g, 200g, 400g, and 500g) have been applied to the device at 3.5 cm on the PFM probe and the resulting output measurements as a function of force is shown in Fig 6. The weights have been chosen accordingly to present the possible expected output force range based on previous studies.

### B. Linearity

**FIGURE 7.**
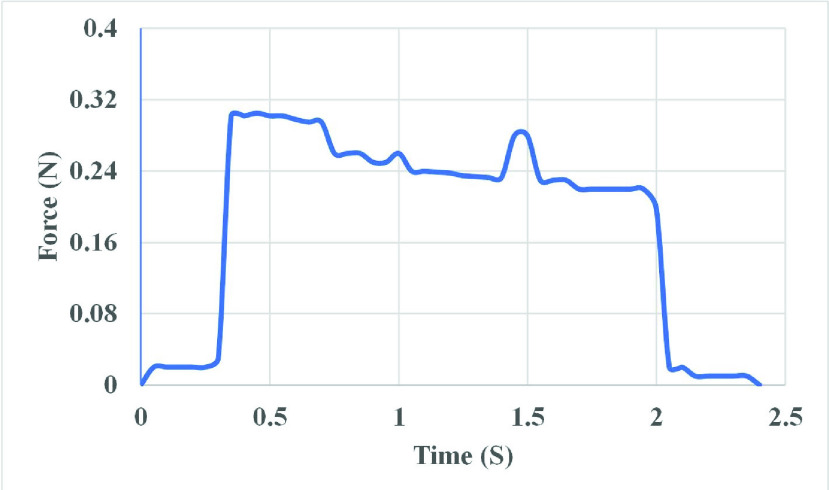
Real-time force variations as a function of time (s) displayed with Arduino IDE software.

The vaginal dynamometer exhibits a linear performance. Linear regression analysis is used in Fig. 6 to compute the slope and intercept factors. The coefficient of determination is calculated and found to be $\text{R}^{2} =0.999$, which corresponds to high linearity. The linear regression equation of the curve is computed to be:

\begin{equation*} \text {Y} = 10.4\text {X} - 0.01\tag{1}\end{equation*}

where Y represents the force (N) and X represents the system output readings. After the calibration is accomplished, the calibration factor is computed, and the real-time force curves are then drawn. Figure 7 shows a recording of pelvic floor maximal strength using the vaginal dynamometer for a participant during the study.

### C. Repeatability

**FIGURE 8.**
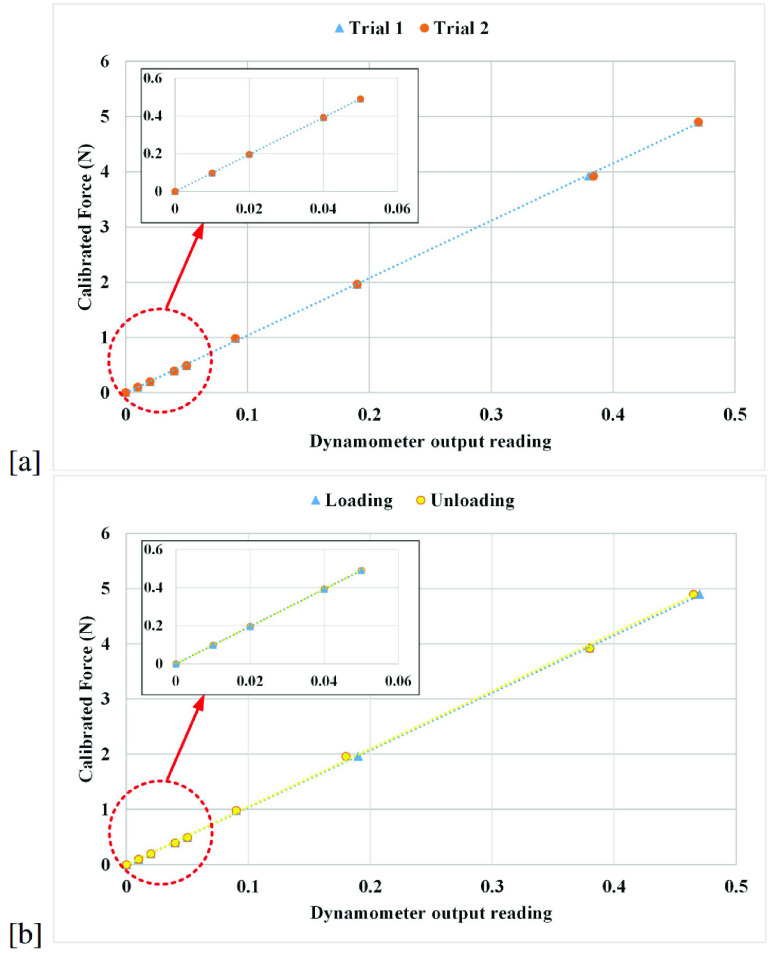
(a) Vaginal dynamometer repeatability graph, and (b) Vaginal dynamometer hysteresis graph.

To evaluate the repeatability of the vaginal dynamometer, the loading technique used in calibration was repeated twice with the same 8 loads previously mentioned at a location of 3.5 cm on the PFM probe. In each trial, the linear regression equation was computed. Figure 8(a) shows the trials 1 and 2 plots obtained. Calculating the mean square error (MSE) for both trial 1 (MSE = 0.003) and trial 2 (MSE = 0.003) with respect to the linear regression equation of trial 1 was similar. Since the linear regression equation obtained from trial 1 predicts well trial 2, the repeatability of the proposed system is validated.

### D. Hysteresis

The hysteresis of the vaginal dynamometer was measured through two load trials at 3.5 cm on the PFM probe. The first load trial is performed with increasing loads, whereas the second load trial is performed with decreasing loads. The same 8 loads (10g, 20g, 40g, 50g, 100g, 200g, 400g, and 500g) have been used in the two trials. A comparison between each load value is conducted. Figure 8(b) shows the hysteresis graphs between the loading and unloading trials. Calculating the mean square error for both the loading trial and the unloading trial with respect to the linear regression equation yielded MSE = 0.003 and MSE = 0.007 respectively. The MSE confirms that the difference between loading and unloading trials is almost negligible.

### E. Noise Effect on System Measurements

The noise effect on the vaginal dynamometer was measured through taking 100, 500, and 1000 recordings for each load consequently. A total of 8 loads (10g, 20g, 40g, 50g, 100g, 200g, 400g, and 500g) are used in this trial. The standard deviation and variance for each load value at 100, 500, and 1000 recording points are calculated and presented in Table 2.

**TABLE 2:** Noise Effect on the System Measurements in Terms of Standard Deviation and Variance Values

Mass (g)		0	10	20	40	50	100	200	400	500
Force (N)		0	0.098	0.196	0.392	0.49	0.98	1.96	3.92	4.9
Noise effect on WPFMM system	100 points STD	0	0.0007	0.0002	0.0004	0.0003	0.001	0.001	0.0023	0.0023
	500 points STD	0	0.0006	0.0002	0.0004	0.0003	0.001	0.0009	0.0023	0.0023
	1000 points STD	0	0.0007	0.0002	0.0004	0.0003	0.001	0.0009	0.0023	0.0023

### F. Power Consumption

**FIGURE 9.**
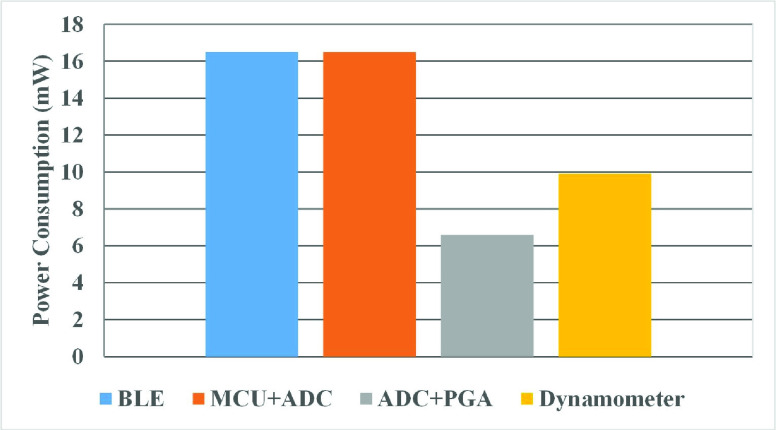
Vaginal dynamometer power distribution across different blocks.

The power consumption of the vaginal dynamometer is evaluated. The system achieves a power consumption of 15mA/49.5mW in operating mode. Figure 9 shows the power consumption breakdown for the system.

### G. Heat Effect on System Measurements

Finally, to evaluate the heat effect on the system, two load trials with the same 8 loads (10g, 20g, 40g, 50g, 100g, 200g, 400g, and 500g) are measured. The first load trial is performed directly after turning the system on ( $\text{t}=0$), then the second load trial is performed after one hour from the initial trial (t = 1 hour) with the system being on for a complete hour duration. The thermal reliability of the system is calculated by running a Cronbach alpha test. The resultant alpha value is 1, which indicates very good reliability. Thus, the effect of the heat on the system measurements is negligible.

### H. Prototypes Variations

Different prototypes of the proposed vaginal dynamometer were produced in preparation for a feasibility study. It was very imperative to study the variations between the different prototypes in terms of linearity, repeatability, and accuracy. The accuracy of each prototype was calculated through comparing the output force (N) of the vaginal dynamometer with the actual force applied (N) from the calibrated weights. Table 3 shows the parameters evaluated with the corresponding prototype.

**TABLE 3:** Final Device Bench Testing Using the Entire System of Prototypes

Test			linearity	Repeatability		Accuracy
			Coefficient of Determination	p-value	t-test	Average %
Dynamometer Prototype Outcome	Dynamometer Prototype	Red	0.995	p >0.05	Not significant	96.2
		Transparent	0.999			96.33
		Yellow	0.999			97.63
		Purple	0.997			97.44
	Average		0.998	difference between Trials 1 & 2 for all prototypes		96.9
	Stand Dev.		0.002			0.739
	Variance		3.67E-06			0.546

## V. Discussion

The proposed vaginal dynamometer utilizes an optimized PFM probe design based on a literature review, four expert physiotherapist evaluations, and a usability study with ten incontinent women.

The PFM probe’s diameter directly affects its usability. Dynamometers with adjustable apertures can accommodate different populations, unlike dynamometers with fixed diameters. For example, nulliparas or women with vaginal atrophy have smaller vaginal openings compared to parous women with levator ani defect. Thus, dynamometers with fixed diameters may not be proper for all women. With the two sizes available for the PFM probe, it is feasible to accommodate women with different hiatus sizes.

Noticeably, the PFM probe design is neither handheld nor fixed to a base, it is meant to stay in place which would presumably allow for more functional assessment conditions (e.g., standing position). Because UI and pelvic organ prolapse symptoms usually occur in the standing position rather than in the supine, it may be of interest that the unit is portable to allow measurements of intravaginal forces in functional positions (such as standing) but has yet to be evaluated in future studies.

The PFM probe is uniquely designed to allow for stabilization inside the vaginal cavity. The side elements of the PFM probe prevent the PFM probe from turning or falling down. Maintaining the PFM probe stability inside the vagina in both standing and supine position is a critical factor in increasing the patients’ comfort during assessment and training sessions. It also insures the validity and repeatability of the measurements.

Another advantage of the device is its lightweight, especially since UI and other lower urinary tract symptoms are more prevalent in the elderly female population, where weight tolerance is a major concern. In addition, the agile methodology used in the production of the vaginal dynamometers allowed for quick and easy iterations throughout the complete production process.

Cleaning and sterilization of the newly designed vaginal dynamometer is one topic to further investigate. With the medical grade silicone material constraint in terms of sterilization, new cleaning and sterilization protocol is sought. Although the number of prototype units used for this study was limited, large-scale fabrication of the device would allow providing a personal device to each patient at a low cost.

Using a computer user interface and a companion app were both required in the design process. The computer user interface allowed the user to visualize the real-time variation of the PFM force measurements, control system characteristics such as the sampling rate, and create a database for the bench test data. Further, the free access DSD TECH Bluetooth mobile application, developed by DSD TECH, was important for the usability study. The physiotherapist used the DSD app as a reference for the vaginal dynamometer functionality. Both GUIs are advantageous in terms of being compatible with any operating system (Microsoft Windows, Linux, macOS/ iOS, and Android).

With a 3.7V/1200 mA Li-Ion battery, and a system power consumption of 49.5 mW at a sampling rate of 20 measurements per second, for 1 h per day (2 sessions of 30 minutes training), 80 days of measurement can be achieved without the need for charging the battery. Taking into account that the vaginal dynamometer is intended to be used for 20-30 minutes, once a day non-consecutively, the heat effect has been evaluated for one continuous hour of use and found to be non-significant. Thus, the possible effect of heat produced by the system components on measurements is non-significant as well.

A communication range of 100 m is achieved by the system in the air. Since wireless communication is between the processing unit and the GUI, the communication range is expected to be high enough to be used at home with a computer, phone, or tablet in the same room.

The vaginal dynamometer prototypes variability is assessed and proven to be non-significant. All the prototypes exhibited a highly linear behavior with a coefficient of determination varying between 0.995 to 0.999. As for repeatability, no significant difference was found between trials 1 and 2 for all vaginal dynamometers. Further, the system average accuracy error lies within 3.9 % of the full scale. Taking into account the evaluation system of the PFM function, the error does not affect the correct categorization or classification of women’s PFM strength and is thus non-significant. Therefore, all the vaginal dynamometer-produced prototypes can be randomly used with confidence in their proper functionality in future studies.

Acknowledging the importance of evaluating the psychometric properties of the system, there is an ongoing feasibility study. The usability of the system is being re-tested as well, in the feasibility study. Future studies are yet to evaluate the effect of intra-abdominal pressure on the system’s measurements, and the feasibility of using the system within different clientele (such as pregnancy and obesity) or with different conditions such as pelvic organ prolapse.

## VI. Conclusion

This paper presented a fully functioning prototype of a portable vaginal dynamometer for the evaluation and training of women with PFM dysfunctions. The developed system allows patients to reproduce natural PFM function with more portability and flexibility. Also, it allows measuring PFM forces in the standing position, which is the naturally occurring position of UI. The system has been tested in a usability study and optimized based on the feedback of participating women. As a second step towards validating the system, the vaginal dynamometer is being used in an ongoing international feasibility study, where it is combined with other sensors and a newly designed exergame for the treatment of geriatric UI. Furthermore, the next version of the proposed system is currently being designed to fully integrate all signal processing and wireless communication electronics into the PFM probe itself.
